# Hepatitis C virus replicons: dinosaurs still in business?

**DOI:** 10.1111/j.1365-2893.2008.01066.x

**Published:** 2009-01

**Authors:** I Woerz, V Lohmann, R Bartenschlager

**Affiliations:** Department of Molecular Virology, University of HeidelbergHeidelberg, Germany

**Keywords:** HCV cell culture system, HCVcc, hepatitis C virus, replicon system

## Abstract

Since the molecular cloning of the hepatitis C virus (HCV) genome for the first time in 1989, there has been tremendous progress in our understanding of the multiple facets of the replication cycle of this virus. Key to this progress has been the development of systems to propagate the virus in cell culture, which turned out to be a notoriously difficult task. A major breakthrough has been the construction of subgenomic replicons that self-amplify in cultured human hepatoma cells. These RNAs recapitulate the intracellular steps of the HCV replication cycle and have been instrumental to decipher details of the RNA amplification steps including the identification of key host cell factors. However, reproduction of the complete viral replication cycle only became possible with the advent of a particular molecular HCV clone designated JFH-1 that replicates to very high levels and supports the production of infectious virus particles. The availability of this new culture system raises the question, whether the use of replicons is still justified. In this review, we will discuss the pros and cons of both systems.

## Introduction

The hepatitis C virus (HCV) is an enveloped positive-strand RNA virus of the genus *Hepacivirus* within the family Flaviviridae [1]. Primary infections with HCV are often asymptomatic or associated with only mild symptoms, but they mostly become persistent causing chronic liver disease [2,3], which may lead to liver cirrhosis and hepatocellular carcinoma. At present, more than 170 million people suffer from chronic hepatitis C [4] making HCV infection a global health problem. Current treatment consists of a combination of polyethylene glycol-conjugated interferon-alpha and ribavirin [4], but patients have to endure serious side effects (reviewed in Ref. [5]). In addition, success rates are limited and the outcome of therapy very much depends on the genotype of the infecting virus [6].

After the first molecular cloning of the HCV genome in 1989 [7], the development of specific antiviral therapies appeared to be within reach. However, progress in drug development was slowed down very much by the lack of systems to propagate HCV in cell culture (HCVcc). Hopes to establish such systems were high with the construction of infectious molecular HCV clones [8,9], but their replication competence demonstrated in experimentally inoculated animals could not be confirmed in cultured cells (reviewed in Ref. [10], see Table 1).

**Table 1:** *In vivo* and *in vitro* replicative capacity of some molecular HCV clones (adapted from Ref. [81])

		*In vivo* study			Cell culture study	
HCV isolate		Infectivity*	References	RNA replication (replicon)	Production of infectious virus	References
H77	1a	+	[8]	+ (requires adaptive mutations)	n.r.	[21]
H77C	1a	+	[9]	+ (requires adaptive mutations, H77S)	±(H77S)	[82,65]
HCV-1	1a	+	[83]	−	n.r.	[84]
BK	1b/1a 3′end†	+	[85]	+ (requires adaptive mutations)	n.r.	[85]
HC-J4	1b ORF/1a 3′NTRs†	+	[86]	+ (requires adaptive mutations)	n.r.	[87]
HCV-N	1b	±	[88]	+ (insertion in NS5A confers adaptation)	−	[77,22,89]
HCV-CG1b	1b	+‡	[90]	+ (DNA-expression system)	+ (DNA-expression system)	[91,92]
Con1	1b	+	[24]	+ (more efficient with adaptive mutations)	±(transient assay wt Con1) − (adapted Con1)	[93,11], T. Pietschmann and R. Bartenschlager, unpublished results
HC-J6(CH)	2a	+	[94]	−	n.r.	[66]
JFH-1	2a	+‡	[28]	++	+ (more efficient with adaptive mutations)	[26,33,28,29]
JFH-1 based chimeras	See Fig. 1B a	+ (J6-JFH-1)‡	[27]	++	++ (J6-JFH-1), + (Con1-JFH-1), ±(H77-JFH-1), ±(452-JFH-1)	[33,30,31]

n.r., not reported.

* Infectivity was tested by inoculation of *in vitro* transcripts into the liver of chimpanzees or mice with human liver xenografts with cell culture grown HCV.

† Chimeric HCV genomes.

‡ Only cell culture grown virus was tested for infectivity.

## HCV replicons of the first generation

A major step forward towards establishment of a robust and reliable cell culture system for HCV was therefore the development of subgenomic and selectable HCV replicons [11]. By definition, a replicon is a nucleic acid (which can be DNA or RNA) that is capable of autonomous replication. In this sense, the HCV genome itself can be regarded as a replicon. However, for clarity, in this review, we refer to replicons as RNA molecules capable only of intracellular self-replication, i.e. unable to support the production of infectious HCV particles.

**Fig. 1:**
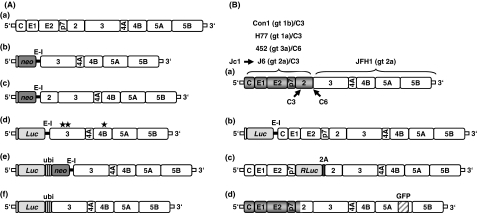
(A) Structures of hepatitis C virus (HCV) replicons. (a) Schematic presentation of the HCV genome. 5′ and 3′NTR are indicated by white bars. (b) and (c) Structures of bicistronic selectable subgenomic replicons. They are composed of the HCV 5′NTR directing translation of the *neo* gene, the IRES of the encephalomyocarditis virus (E-I), the HCV replicase genes NS3–NS5B (a) or NS2–NS5B (b) and the 3′NTR. (d) Structure of a subgenomic Con1 reporter replicon that is used for transient replication assays. Shown is a luciferase (*Luc*) reporter replicon that contains three cell culture adaptive mutations (indicated with asterisks) to enhance RNA replication [14]. (e) Structure of a selectable reporter replicon. This construct encodes a luciferase–ubiquitin–neomycin phosphotransferase fusion protein in the first cistron. The RNA supports stable expression of luciferase; ubiquitin (ubi) is used to trigger proteolytic removal of the neomycin phosphotransferase from the fusion protein. (f) Structure of a monocistronic reporter replicon. A gene encoding luciferase is fused in frame to the viral replicase genes NS3–5B via ubiquitin. The entire polyprotein is translated by the HCV IRES. (B) Structure of JFH-1 derived genomes. (a) Chimeric genomes are composed of the region encoding core to transmembrane segment 1 of NS2 (C3 position) of HCV isolates Con1, H77 or J6 fused to the remainder of the JFH-1 isolate. In case of the 452 chimera, the region encoding core to the C-terminus of NS2 (C6 position) was fused to the JFH-1 replicase genes. Junction sites are indicated by an arrow. The highly assembly competent J6/JFH-1 chimera is also called Jc1 [31]. (b) Schematic representation of a bicistronic full length JFH-1 reporter genome. The HCV 5′NTR directs translation of the firefly luciferase gene (*Luc*) whereas translation of the HCV polyprotein is under the control of the EMCV IRES (E-I). (c) Schematic diagram of a monocistronic Renilla luciferase (RLuc) reporter genome [38]. (d) Structure of a monocistronic Jc1 reporter virus applicable for live cell imaging. The gene encoding the green fluorescent protein (GFP; striped box) is inserted in-frame into domain 3 of NS5A [40].

Key to the establishment of the HCV replicon system was a molecularly cloned full length genome, designated Con1, in which the region encoding for the proteins core to nonstructural protein 2 (NS2) or p7 was replaced by two nonviral elements: first, the neomycin phosphotransferase (*neo*) gene that allows to select for resistance against the drug G418 and second, the internal ribosome entry site (IRES) from a picornavirus (Fig. 1A a,b,c). The resulting construct was bicistronic with the first cistron encoding the neo gene and the second, the HCV replicase genes (NS3–NS5B). Upon transfection of the human hepatoma cell line Huh-7 with *in vitro* transcripts derived from the cloned replicon cDNA and subsequent G418 treatment, it became possible to select for a few G418-resistant colonies that supported HCV RNA replication to unexpectedly high levels (1000–5000 positive-strand RNA molecules per cell). Replication of these RNAs was resistant against actinomycin D which blocks DNA-dependent, but not RNA-dependent RNA synthesis thus providing formal proof that the HCV replicons indeed replicated autonomously in these cells.

Subsequent to the establishment of Con1 replicons, the same approach was used to generate replicons of other HCV isolates and nowadays replicons derived from more than eight isolates and covering genotypes 1a, 1b and 2a are available (Table 1).

## Improvements of the replicon system

Further studies identified two major prerequisites for efficient replication of these HCV replicons: first, the selection for replication-enhancing (cell culture adaptive) mutations; second, the selection for particular Huh-7 cells that are highly permissive for HCV RNA replication [12]. Adaptive mutations were found to cluster in four distinct regions of the HCV polyprotein: in the N-terminus of the NS3 helicase, at two positions in NS4B and in the centre of NS5A. Recloning of these mutations into the parental replicon resulted in a strong increase in RNA replication efficiency with the degree of enhancement depending on the particular mutation or combination of mutations [13–15]. Interestingly, adaptive mutations at very similar or even identical positions were found in all replicons based on HCV isolates of genotype 1 established so far, indicating that the mechanisms underlying cell culture adaptation is conserved within this genotype (Table 1). The second determinant for efficient RNA replication turned out to be the selection for highly permissive cells [14,16,17]. In a given Huh-7 pool, only a few cells appear to support HCV RNA replication to a high level and G418 selection results in an enrichment of these permissive cells. Replicon RNAs can be removed from such cell clones by drug treatment resulting in so-called ‘cured’ Huh-7 cells that support HCV RNA replication to a higher level as compared with the parental cells. Two prominent examples of such cell clones are Huh7.5 and Huh7-Lunet [16,18].

The availability of highly permissive cell clones and adaptive mutations greatly broadened the scope of HCVcc systems. For instance, reporter replicons containing the luciferase gene and applicable for transient (short-term) assays became available (Fig. 1A d) [13,14]. In addition, cell lines containing stably replicating reporter replicons were generated that are well applicable for high-throughput screening purposes (Fig. 1A e) (reviewed in Ref. [19]). Monocistronic replicons were constructed as well, containing only the HCV IRES and thereby closely mimicking the translational properties of authentic viral genomes (Fig. 1A f) [20]. Moreover, it became possible to generate stable cell clones carrying self-replicating genomic replicons expressing all viral proteins [16,21–23]. However, attempts to demonstrate HCV particles released into the culture supernatant of these cells failed. This is due to the fact that cell culture adaptive mutations interfere with the production of infectious virus particles (T. Pietschmann and R. Bartenschlager, unpublished results). In line with this observation, a Con1 genome containing such adaptive mutations was unable to establish productive infection *in vivo* whereas chimpanzees inoculated with the wild type Con1 genome were readily infected [24].

## The infectious virus system (HCVcc)

Inspite of great progress with HCV replicons, the lack of virus production was an obvious limitation of this system. Because of the assembly block imposed by adaptive mutations on one hand, and the very low (most often nondetectable) replication of nonadapted replicons on the other hand, a full-cycle culture system only became possible with the identification of an HCV isolate that was capable of high-level replication without requiring cell culture-adaptive mutations. This ‘holy grail’ was discovered by Kato et al. [25], who cloned a genotype 2a consensus genome designated JFH-1 from a Japanese patient with fulminant hepatitis. For reasons still not understood, replicons derived from this isolate replicate to exceptionally high levels without requiring adaptive mutations [26]. Most importantly, transfection of JFH-1 full-length genomes into Huh-7 cells supports the production of virus particles that are infectious in cell culture and inoculated animals (chimpanzee and transgenic mice with human liver xenografts), confirming that cell culture-derived HCV particles (designated HCVcc) represent authentic virions [27–29].

## Improvements of the HCVcc system

In the light of the rather low infectivity titres attained with JFH-1 [about 10^4^ tissue culture infectious dose 50 (TCID_50_) per mL], much effort was made to improve the system. One major progress was the generation of virus chimeras consisting essentially of the JFH-1 replicase (NS3–NS5B) fused to the core to NS2 region of other HCV isolates (Fig 1B a). Lindenbach *et al.* [30] described a chimera consisting of the structural region of a genotype 2a isolate [J6 (CF)] fused to the JFH-1 replicase. Pietschmann and colleagues identified a more efficient fusion site (designated ‘C3’) and enlarged the panel of virus chimeras to the isolates Con1 (gt 1b), H77 (gt 1a), 452 (gt 3a) and J6 (gt 2a) (Fig.1B a). Virus titres of the most efficient intragenotypic chimera that was designated Jc1 are in the range of 10^6^ TCID_50_/mL [31], i.e. about 100-fold higher compared with JFH-1.

Alternative to virus chimeras, cell culture adaptation of JFH-1 by continuous passage of persistently infected cells allowed the construction of highly efficient JFH-1 variants that also support virus production to much higher levels as compared with the parental JFH-1 [29,32–35]. Finally, the identification of proper Huh-7 cell clones was again a key for a robust HCVcc system as it was for the replicons. Two notable examples are Huh7.5 and Huh7-Lunet/CD81 high. The latter are derived from Huh7-Lunet cells by stable over-expression of CD81, which is required for efficient infection [36]. Recently, a novel human hepatoma cell line, LH86, was shown to be susceptible to HCV infection and supporting HCV RNA replication [37].

In analogy to HCV replicons, JFH-1 derived virus genomes with insertions of reporter genes have been described (Fig.1B b,c) [28,38,39]. Moreover, genomes with a stably inserted gene encoding for the green fluorescent protein were generated allowing the live visualization of HCV infected cells [40] (Fig. 1B d).

## Other cell-based HCV systems

**Fig. 2:**
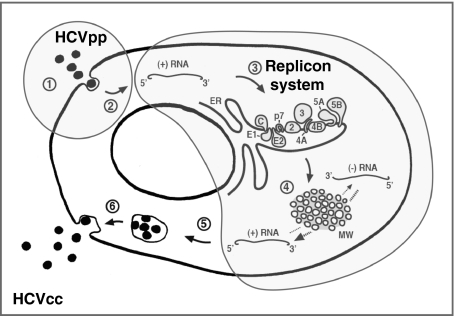
Schematic representation of the HCV replication cycle and steps that can be addressed with the various cell culture systems. Upon binding to a permissive host cell [73], HCV particles enter the cell most likely by receptor-mediated endocytosis [62]. The RNA genome is liberated into the cytoplasm and translation occurs at the ER membrane [19]. Upon processing of the polyprotein, the membranous replication complex is formed (membranous web; MW). [10] This complex is the site of viral RNA amplification. Positive strand RNA progeny is encapsidated [12] and virus particles appear to be released from the cell by the constitutive secretory pathway [81]. Steps of the replication cycle that can be studied with HCVpp, replicons or the HCVcc system are indicated by shaded areas or the outer box respectively. Scheme from Darius Moradpour with slight modifications.

Amongst the other available model systems to study HCVcc, the pseudoparticle system (HCVpp) is perhaps the most important one [41,42] (Fig. 2). HCVpps are retroviral nucleocapsids surrounded by a lipid envelope that contains authentic HCV glycoprotein complexes. As the early steps of the viral replication cycle are driven by the envelope proteins largely independent from the other structural proteins, HCVpp is an ideal system to study receptor binding and entry and has been used intensively to characterize neutralizing antibodies.

Very recently, Steinmann et al. [43] described the production of HCV-like particles (referred to as HCV_TCP_) that are composed of authentic virus particles that contain a subgenomic RNA. Upon infection of Huh-7 cells, these RNAs replicate to high levels, yet do not produce infectious progeny because of the deletion of the structural genes. This system is also well suited to study the early and late steps of infection, but does not require a biosafety level 3 laboratory as is the case for full-length HCV sequences.

Another system to study the HCV replication cycle in a more authentic setting is the infection of primary human hepatocytes (PHH). Successful infection could be demonstrated with serum- and cell culture-derived HCV particles [44,45]. However, preparation of PHH is a time-consuming and laborious process, the quality of PHH is difficult to control and genome replication of JFH-1 viruses is about 1 order of magnitude lower in PHH compared with Huh-7.5 cells [45].

Prior to the establishment of HCV replicons, surrogate models have been used, in particular, the pestivirus bovine viral diarrhoea virus (BVDV) that shares many features with HCV such as the genome organization and the overall replication cycle. As BVDV can easily be cultured *in vitro*, it has been a promising model system (reviewed in Ref. [46]). An additional surrogate model is based on GB virus B (GBV-B), one of the closest genetic relatives to HCV within the Flaviviridae family. GBV-B replicons have been constructed that are similar to those developed for HCV, but propagation of infectious GBV-B is also only possible in primary cell cultures (reviewed in Ref. [47]).

## The pros and cons of the HCV replicon and the HCVcc system

With the plethora of cell-based HCV replication systems developed in the past few years, most notably the HCVcc system, the question arises as to which role replicons will play in the future both in basic and applied research. At a first glance, the HCVcc system is superior because it covers the complete viral replication cycle (Fig. 2). However, the early steps (virus binding, entry, uncoating) can already be addressed with the HCVpp system (Fig. 2). In fact, most, if not all observations made with the HCVpp system have been confirmed by using cell culture grown HCV particles. For instance, CD81 and scavenger receptor B class I have been validated as important entry molecules (reviewed in Ref. [48]). Moreover, the essential role of claudin-1 in HCV entry was recently identified by using HCVpp and verified by HCVcc [49]. Finally, HCV entry via a low pH-dependent step originally described with HCVpp has now been confirmed with HCVcc [39,42,50]. Nevertheless, because of the higher authenticity, most future studies will analyse HCV infection primarily by using cell culture grown HCV. Moreover, when using a highly assembly competent HCV variant, the production of high titre HCVcc stocks is easier compared with HCVpp stocks, which cannot be passaged but rather must be generated by repeated DNA transfection.

When considering RNA replication (Fig. 2), results obtained with the HCVcc system or the replicon system will likely be the same. For instance, the important role of lipids for RNA replication observed with subgenomic replicons has been confirmed in the HCVcc system [51,52]. Likewise, the dependence of HCV replication on cyclophilins was found both in the replicon system and with HCVcc [53–55]. Depending on the particular question, one system may be superior over the other. For instance, when performing knock-down experiments of a particular host cell factor, the impact of the knock-down often is easier to detect in an infection-based system rather than in the replicon system. This is due to the fact that upon infection, only few virus genomes enter the cell, especially when using a low multiplicity of infection. In this setting, a limiting host cell factor will impose a much higher barrier to RNA amplification compared with cells with a stable replicon where the RNA copy number is already high and where stable replication complexes have already been formed. Still, replicons will remain indispensable to analyse the mechanistic roles of host factors, NS proteins and *cis*-acting elements in RNA replication compared with other stages of the viral life cycle.

Probably, the strongest impact the HCVcc system has deals with the late steps of the replication cycle, i.e. assembly and release. These aspects could not be addressed properly by any of the surrogate systems. Although the formation of virus-like particles had been described in insect cells upon infection with recombinant baculoviruses or in mammalian cells upon transfection with alphavirus vectors directing the expression of the HCV structural genes, none of these systems adequately reflects authentic HCV assembly [56,57]. This is best illustrated by the fact that in none of these systems, virus (-like) particles were secreted. In contrast, infectious particles are readily assembled in the HCVcc system and some variants support high titres. Taking advantage of that system, it soon became clear that HCV particles assemble in close proximity of the surface of lipid droplets where the majority of core protein accumulates [58–60]. Infectious virus particles appear to acquire their envelope by budding into the lumen of the endoplasmic reticulum in close apposition to lipid droplets and are released from the cell in close association with the very-low-density lipoprotein pathway [61]. Apart from these insights into the peculiarities of HCV assembly, the possibility to produce infectious virus particles in the laboratory under well-defined conditions opens new avenues to visualize infectious HCV. In this respect, there is no alternative to the HCVcc system. However, increasing evidence points to important roles of HCV NS proteins, particularly NS5A, in viral assembly and subgenomic replicons will remain valuable tools to dissect the different functions of these proteins [33,62–64].

The major limitation of the HCVcc system is its restriction to a single isolate (JFH-1) that supports efficient virus production. Although, production of infectious particles has also been described for a highly adapted H77 (genotype 1a) isolate, virus titres attained with this genome and infectivity of released particles appear to be very low [65]. In that respect, HCV replicons cover a broader range of isolates and genotypes (Table 1), particularly of genotype 1, which is most common worldwide. The availability of such a spectrum of systems is probably most important for the development of antiviral drugs. In fact, subgenomic HCV replicons contain the prime drug targets: the NS3 protease and the NS5B RdRp. Moreover, replicons are more amenable for genetic manipulation such as the construction of chimeras in which the protease or polymerase gene is replaced by the corresponding gene from another HCV isolate or even from genomes isolated from an infected patient. In contrast, work in our laboratory has shown that replacing, e.g. the NS3 protease gene of JFH-1 by the one of another functional HCV clone in all cases very much reduced or completely abolished virus assembly (A. Kaul, A.L. Gamer and R. Bartenschlager, unpublished results). Likewise, as the NS5B gene of JFH-1 appears to be a major determinant for efficient RNA replication, replacements by NS5B genes from other HCV isolates are very difficult [20,66]. In contrast, such replacements have been described for subgenomic replicons derived, e.g. from Con1 and utilized to established phenotypic resistance assays [67,68].

Because of the high ‘genetic flexibility’ of replicons, they can be designed with much more ease as compared with full length virus genomes. For instance, selectable replicons with a stably expressed reporter gene (Fig. 1A e) have been used for high throughput drug screening purposes (reviewed in Ref. [19]). Because of the easy read-out and the robust signal, such assays can be automated and adjusted to robotics, which is important not only for drug screening but also for screening of large-scale small interfering RNA libraries in search for host cell factors required for HCV replication [69,70]. Moreover, replicons do not impose a biohazard allowing screening purposes under the lowest biosafety levels, which is not possible when working with the HCVcc system. In case of the latter, a high throughput screen will also require the prior large-scale production of infectious virus stocks, which may be challenging even when using highly assembly competent variants such as Jc1.

Hepatitis C virus replicons have also been very instrumental to select for antiviral drug resistance. In most cases, selectable replicons have been used in which the selection marker (e.g. G418 for *neo* replicons) is exploited to preserve the replicon in the face of selective pressure imposed by an antiviral compound, resulting in the selection of cell clones with conserved mutations in the viral RNA. In contrast, when using the HCVcc system, this is not really possible because a strong antiviral compound with a high genetic barrier will rapidly eliminate the virus from the culture (A. Kaul and R. Bartenschlager, unpublished results). Furthermore, if mutations at independent positions confer resistance, it will be hard to identify relevant mutations in the viral quasispecies. Although the construction of bicistronic selectable JFH-1 genomes that support the production of infectious virus particles is possible [71], replication of such genomes is extremely impaired and virus titres are very low. Therefore, such genomes will be rapidly lost when passaging cells in the presence of an antiviral compound (A. Kaul and R. Bartenschlager, unpublished results).

Possible limitations originally described for HCV replicons are the restriction to Huh-7 cells and the dependence on host cell growth. With respect to cell lines, stable replication (by using selectable replicons) has now been achieved for a number of other cell lines such as human hepatoma cells (HuH6 or LH86 [37,72]), human nonliver cells (HeLa or 293 [73–75]), nonhuman liver cells (Hepa1–6 [75]) and nonhuman nonliver cells (mouse embryonic fibroblasts [76]). Moreover, the inhibition of HCV replication that was observed in confluent nondividing Huh-7 cells has not been found in HuH6 [72] and HeLa [77] cells. The underlying reasons are not quite clear, but may be because of limiting nucleoside triphosphate pools in resting Huh-7 cells [78]. It should be pointed out that the same observations have been made when using JFH-1 derived replicons and the full length genome. In fact, virus production also ceases in confluent Huh-7 cells and efficient replication and spread is largely restricted to particular Huh-7 cell clones. Thus, these limitations are no peculiarities of certain replicons, but rather reflect some properties of HCV replication in certain host cells.

## Perspectives

There is no doubt that the possibility to produce recombinant infectious HCV particles in the laboratory opens new avenues to study the viral replication cycle. The early and late steps can now be targeted by inhibitors of virus binding and fusion [79] or assembly and release [80]. The construction of virus chimeras composed of the JFH-1 replicase and the structural region of other isolates has broadened the scope of the HCVcc system, which is particularly helpful when studying neutralizing antibodies [28]. However, a major limitation is the restriction to a single genotype 2a isolate, which from a drug development point of view is least interesting because of the rather high sustained viral response rates to current therapy of patients infected with genotype 2 viruses. Construction of replicase chimeras so far were of limited success and further studies will be required to overcome that hurdle and to identify novel molecular HCV clones replicating to high level in cell culture and supporting the production of infectious particles. At least until then, but most probably beyond this time point, the replicon system will be a valuable and important tool for basic and applied research.

## Abbreviations:

BVDV: bovine viral diarrhoea virus
HCV: hepatitis C virus
HCVcc system: HCV cell culture system
HCVpp system: HCV pseudoparticle system
Huh: human hepatoma cell line
JFH: Japanese patient with fulminant hepatitis
NS2: nonstructural protein 2
PHH: primary human hepatocytes
